# Individual Variability in Physiological Responses and Psychological Conditions Associated With Methamphetamine Use: Pilot Ecological Momentary Assessment Study Using a Wearable Device and Self-Monitoring Mobile App

**DOI:** 10.2196/73790

**Published:** 2026-03-02

**Authors:** Ayumi Takano, Kayo Okuda, Jun Sese, Koki Ono, Toshihiko Matsumoto

**Affiliations:** 1 Department of Drug Dependence Research, National Institute of Mental Health, National Center of Neurology and Psychiatry, 4-1-1 Ogawa-Higashi, Kodaira, Tokyo, 187-8553, Japan, 1 42-346-1861; 2 Humanome Lab, Inc, Tokyo, Japan; 3 Department of Preventive Medicine and Public Health, School of Medicine, Keio University, Tokyo, Japan

**Keywords:** methamphetamine, wearable device, physiological data, mobile app, ecological momentary assessment, self-monitoring, digital therapeutic intervention

## Abstract

**Background:**

Digital mental health approaches offer a novel means to monitor and reduce harms associated with substance use in daily life. However, limited evidence exists on their application for methamphetamine (MAMP) use, particularly regarding individual variability in physiological responses and psychological conditions.

**Objective:**

This pilot study aimed to explore inter- and intraindividual differences in craving, emotion, and heart rate associated with MAMP use, using data collected from a wearable device (Fitbit) and a mobile-based self-monitoring app.

**Methods:**

Participants were individuals with MAMP use disorder receiving outpatient treatment in Japan. The analysis included 7 participants who used MAMP during an 8-week observation period. Physiological data, including heart rate and sleep patterns, were collected using Fitbit devices, while daily self-reported MAMP use, craving intensity, and emotional status were recorded via a mobile app. After syncing the data, we visualized and summarized individual MAMP use patterns in detail. Correlations between physiological and psychological indicators and the frequency of MAMP use per day were analyzed. In addition, heart rate trends before and after MAMP use events were evaluated using a linear mixed effects model, and both interindividual variability and intraindividual variability were assessed.

**Results:**

Patterns of MAMP use varied widely across participants, with it most commonly occurring in the morning or at night, regardless of the day of the week. Craving and negative emotions were frequently reported on MAMP use days and were positively correlated with the number of MAMP use episodes per day. Participants who used MAMP more frequently exhibited relatively higher resting heart rates. Following MAMP use, heart rate increased significantly and remained elevated for up to 9 hours. Sleep duration and frequency were reduced or absent on MAMP use days. Approximately 64% of the variance in heart rate was attributable to interindividual differences, while 12% reflected variability across events within the same individual.

**Conclusions:**

This pilot study demonstrates the feasibility and value of using digital tools to examine physiological responses and psychological conditions associated with MAMP use in real-world settings. Persistent cardiovascular activation and disrupted sleep highlight the potential risks of long-term MAMP use. Individual differences in heart rate responses, craving, and emotional states underscore the importance of personalized intervention strategies. Integrating real-time self-monitoring, notifications for elevated heart rate, and online cognitive behavioral therapy into digital therapeutic interventions may improve health outcomes for individuals with MAMP use disorder.

## Introduction

Digital mental health is a promising approach to assessing and predicting mental health conditions [1-3], including issues related to alcohol and other drug use [4-8]. It enables the real-time and continuous collection of data in daily life through wearable devices and mobile apps. By leveraging these data, researchers can obtain deeper insights into both objective physiological indicators (eg, heart rate, sleep patterns, physical activity) and subjective, self-reported mental health conditions (eg, mood, sleep quality, substance use, craving). Additionally, researchers can develop predictive models of mental health conditions and intervention strategies. In fact, wearable devices and mobile apps have been used in numerous studies to address issues of mental health and substance use [9-11]. However, digital mental health studies related to substance use are less common than those focused on other mental health issues, and there is a lack of sufficient evidence to validate the effectiveness of remote monitoring of substance use disorders (SUDs) using wearable devices [12,13]. Implementing evidence-based digital health interventions for individuals with SUDs remains challenging [14,15].

When analyzing data from wearable devices and mobile apps, complex analytical methods, such as machine learning, are required depending on the study purpose and characteristics of the data obtained [16,17]. These datasets often contain large volumes of continuous and repeated measurements (ie, minute-by-minute, hourly, or daily data), as well as missing data. Physiological and psychological health indicators change over time and are influenced by an individual’s characteristics. Therefore, addressing both interindividual and intraindividual variability is crucial when analyzing data or developing interventions based on the analysis results. However, standardized methodologies for data analysis and intervention development have not yet been well established [16,18]. More findings from studies in the field of digital mental health are needed to effectively apply complex data to real-world interventions, particularly in the area of substance use.

Moreover, few studies have focused on individuals who use methamphetamine (MAMP), as most research has concentrated on those who use alcohol, tobacco, or cannabis [6,8,19]. MAMP use is becoming more prevalent worldwide [20], with significant effects on the cardiovascular system, sleep, psychotic symptoms, and behavior [20,21]. Analyzing real-world data from individuals who use MAMP is crucial for assessing their physiological and mental health conditions during MAMP use and identifying key features that predict harms associated with MAMP use. Several laboratory-based studies have demonstrated that MAMP administration produces acute increases in heart rate and blood pressure, as well as significant disruptions in sleep architecture, including prolonged sleep latency and reduced REM sleep [21-25]. Self-reported studies among people with MAMP use disorder have similarly shown persistent insomnia and poor sleep quality during abstinence. However, these findings have been largely derived from controlled laboratory or inpatient settings, where ecological validity is limited. In contrast, very few studies have examined real-world physiological changes associated with MAMP use. Existing ecological momentary assessment (EMA) studies have primarily focused on psychological and behavioral dimensions, such as craving, mood, and contextual triggers (eg, Reback et al [26]), but momentary physiological indices, such as heart rate, activity, and sleep patterns, have rarely been captured in daily life. Integrating EMA with wearable sensing technologies therefore represents a promising novel method to elucidate the dynamic interplay between MAMP use, heart rate, and sleep conditions in naturalistic settings. Accordingly, this aimed to combine EMA and wearable physiological monitoring to explore how MAMP use affects real-time fluctuations in heart rate and sleep among individuals in their everyday environments. These insights could be valuable for developing new ecological momentary interventions, such as relapse prevention [27].

This pilot study aimed to preliminarily evaluate detailed patterns of MAMP use and physiological and psychological status when using MAMP and to explore both the interindividual and intraindividual variability in heart rate using physiological data from a wearable device (Fitbit) and self-reported data from a mobile-based substance use self-monitoring app. The findings may contribute to a better understanding of the health effects of MAMP use and will be useful for informing the protocol design of a subsequent larger-scale study.

## Methods

### Participants

Participants were patients with MAMP use disorder receiving outpatient treatment at a psychiatric hospital in Japan. Because MAMP use is illegal in Japan and many people who use it are concerned about the confidentiality of their information, recruiting participants from the general population was considered difficult. Therefore, we targeted individuals receiving outpatient treatment, for whom face-to-face recruitment was feasible. The inclusion criteria were (1) severity of MAMP use within the past year assessed as a score between 1 and 8 on the Drug Abuse Screening Test 10-item version (DAST-10) [28,29], (2) ownership of a smartphone (iOS 14 or later or Android 7.0 or later), (3) ability to wear a Fitbit continuously and to record daily entries using a self-monitoring app for 8 weeks, and (4) age 20 years or older. The exclusion criteria were (1) inability to speak or write in Japanese, (2) lack of internet access or inability to complete web-based surveys, (3) inability to participate for the entire 8-week study period, and (4) determination by the primary physician that study participation was inappropriate.

Psychiatrists specializing in SUDs screened potential participants based on the predefined inclusion and exclusion criteria. All participants were informed of the study aim and procedures and submitted written informed consent. A total of 13 participants were enrolled from September 2021 to April 2022. For this study, we used data from the 7 participants who used MAMP during the study period and with Fitbit data when they used MAMP. We excluded participants (n=6) who did not use MAMP at all during the study period or did not wear a Fitbit device when they used MAMP.

### Devices and Measurements

We previously reported the study protocol [30], providing an overview of the obtained data and preliminary analysis methods. In this study, we conducted a more detailed analysis, focusing specifically on individual differences in physiological responses before and after MAMP use events.

#### Fitbit Data

Fitbit is a wearable activity tracker, designed for use with both Android and iOS smartphones. In this study, Fitbit Inspire 2 [31] was used. To reduce measurement variability, all participants in this study used the same Fitbit Inspire 2 model and were provided with standardized wearing instructions throughout the study period. The device collects data such as heart rate per minute, sleep duration and quality, daily step count, and overall physical activity. Participants were instructed to wear the Fitbit device for 8 weeks and to regularly sync it with their smartphones via the Fitbit app. If a participant had missing data for 3 or more consecutive days, research staff sent reminder emails encouraging them to wear the device and sync it regularly. The synchronized data were retrieved daily from the Fitbit server through an application programming interface (API) and securely stored in a research database developed for this study, with all data anonymized to protect participant privacy.

We assessed two types of heart rate data in this study. The first was daily resting heart rate, as estimated by the Fitbit algorithm. The second was interval-based mean heart rate, which captures the average heart rate across 3-hour intervals before and after reported MAMP use. Specifically, we analyzed 6 time intervals: 6 hours to 9 hours before, 3 hours to 6 hours before, 0 hours to 3 hours before, 0 hours to 3 hours after, 3 hours to 6 hours after, and 6 hours to 9 hours after MAMP use. These intervals were chosen based on the approximate half-life of MAMP, which is approximately 10 hours [32]. By covering up to 9 hours on either side of MAMP use, we aimed to encompass the period during which the stimulant’s physiological effects were likely to persist. Segmenting the data in this way allowed us to observe heart rate fluctuations potentially associated with the acute effects of MAMP.

Sleep condition was also assessed using two metrics: (1) sleep count (the number of sleeps detected by Fitbit in a single day) and (2) total main sleep time (the primary overnight sleep duration, excluding short naps). This latter metric reflects the continuous period from initial bedtime to final awakening.

Additionally, we calculated Fitbit device wearing rates by dividing the total number of minutes during which heart rate data was recorded by the total number of minutes in a day (ie, 1440 min). This measure was used to assess data completeness and to help identify behavioral patterns that may occur when participants remove the device in specific situations, such as during MAMP use.

Fitbit devices have been extensively evaluated in validation studies and systematic reviews, demonstrating acceptable validity for heart rate measurement, particularly under resting and low-to-moderate activity conditions, although slight underestimation compared with criterion measures has been reported [33,34]. Measurement accuracy is known to vary according to activity intensity, device model, and wearing conditions. For sleep assessment, Fitbit devices show high sensitivity for detecting sleep versus wake states but relatively lower specificity for detecting wakefulness [33,35]. As a result, total sleep time and sleep efficiency may be overestimated, while wake after sleep onset may be underestimated when compared with polysomnography [35]. Newer Fitbit models, including the Inspire 2, incorporate combined sensing approaches using accelerometry and heart rate–based algorithms, which have demonstrated improved performance over earlier generations.

In this study, physiological data were collected under an EMA framework in free-living conditions. Unlike biometric measurements obtained under standard circumstances, such as laboratory-based or clinically supervised settings with controlled posture, activity, and timing, the data in this study were captured continuously during participants’ daily lives and in temporal proximity to self-reported events. As a result, collected data reflect real-world physiological states and may include greater variability due to uncontrolled environmental and behavioral factors.

#### Self-Monitoring App Data

Self-monitoring is widely used in the field of substance use and has been shown to effectively reduce drug use [36]. In ecological momentary studies, various types of self-reported data have been assessed, including substance use (use of MAMP and other drugs), craving, and emotions [10,11]. We developed a self-monitoring app for this study. Participants recorded the following data once a day for 8 weeks: (1) type of drug used; (2) start and end times of the drug use; (3) emotion status for the day, selected from 4 common emotional icons (good, not good, sad, angry); and (4) craving intensity, rated on a slider scale ranging from 0 to 10. These data were also stored securely and anonymously in the research database.

We summarized MAMP use patterns to confirm the frequency and timing of MAMP use both across participants and for each individual. Specifically, we examined the total number of days of MAMP use during the 56-day study period, average number of days of use per week, consecutive days of use, weekend use (ie, use on Saturdays and Sundays), the number of uses per day on days when MAMP was used, and the time of day MAMP was used (morning: 6‐12 AM, afternoon: 12‐6 PM, night: 6‐12 PM, midnight: 12‐6 AM). Additionally, we summarized craving intensity and emotional status on days when MAMP was used.

### Analysis

#### Imputation of Missing Data and Noise Correction

Fitbit data were missing when the device was not worn. Heart rate, sleep, and activity data during these periods were treated as missing values. However, for graphical visualization purposes, these missing values were displayed as zero.

Heart rate data often contained noise, potentially affecting its accuracy and interpretation. We identified and corrected two types of noise: transient noise occurring whether the device was worn or not and continuous noise occurring while the device was worn. To address transient noise, we applied a moving average to smooth brief fluctuations in heart rate. Specifically, a 3-minute window was used to average out short-term spikes or drops, preserving the overall trend without overly distorting the data. For continuous noise, we applied a moving maximum with a 5-minute window, which more effectively captured upper boundary values in segments where heart rate readings were consistently unstable.

On days with no app entries, all questions were treated as missing. Even on days with entries, any unanswered questions were also considered missing. If the question regarding MAMP use was missing for a given day, that day was excluded from calculations related to MAMP use.

#### Descriptive Statistics

First, demographic characteristics of the participants, emotional status, craving, heart rate, and Fitbit wearing rate were summarized during the study period. Second, for each participant, patterns of MAMP use, Fitbit wearing rate, emotional status, craving, and heart rate on MAMP use days were calculated and visualized. In addition, physiological data collected by Fitbit during the study period were visualized to examine individual trends. Python (3.10.10), with the pandas (2.2.3), matplotlib (3.7.3), and seaborn (0.12.2) libraries, was used for these analyses and visualizations.

#### Analysis of Interindividual and Intraindividual Differences

To examine the relationship between psychological and behavioral states and MAMP use, both within individual participants and across all participants, we assessed the correlations between the amount of MAMP use on MAMP-use days and emotion, craving, and heart rate using Pearson correlation coefficients. When calculating the correlation coeffficients, the 4 emotional categories were operationally converted into numerical values as follows: good=1, not good=0, sad=–1, angry=–2. These calculations were performed in Python (3.10.10) using the scipy.stats.pearsonr function from scipy (1.15.2).

To examine interindividual differences in heart rate trends before and after MAMP use, we categorized the mean heart rate when using MAMP for each participant into 6 time intervals: 6 hours to 9 hours before, 3 hours to 6 hours before, 0 hours to 3 hours before, 0 hours to 3 hours after, 3 hours to 6 hours after, and 6 hours to 9 hours after. We included only the data collected when heart rate could be monitored for 9 hours before and after MAMP use. Moreover, to examine intraindividual differences in heart rate before and after MAMP use, we calculated the mean heart rate trend for each MAMP use event. A linear mixed effects model was used to evaluate changes in heart rate before and after drug use and to assess interindividual and interevent differences. This model is most appropriate for handling repeated daily measurements nested within individual participants [37]. The interval-based mean heart rate was set as the dependent variable. The time periods (6‐9 h before, 3‐6 h before, 0‐3 h before, 0‐3 h after, 3‐6 h after, and 6‐9 h after) were included as fixed effects. The period 6 hours to 9 hours before drug use was set as the reference category for comparison with other time periods. The participant ID was treated as the primary random effect, and MAMP use events nested within each participant were treated as an additional random effect. Additionally, to account for the autocorrelation among consecutive measurements within each event, a first-order autoregressive model was used. The model was estimated using restricted maximum likelihood and analyzed using the nlme package in R (version 3.1.167). We excluded a participant who did not wear a Fitbit on days of MAMP use from the linear mixed effects model analysis. The statistical significance of differences between time periods was evaluated using *t* tests, with the significance level set at *P*<.05.

To quantify the magnitude of interindividual differences, we examined the variance components of the model and calculated the intraclass correlation coefficient. The proportion of total variance attributable to differences between participants was calculated to determine the extent of interindividual variability in heart rate responses. Similarly, the significance of interevent differences was evaluated by examining the proportion of total variance attributable to event-specific variability within the same individual.

### Ethical Considerations

This study was approved by the Ethics Committee of the Faculty of Medicine and Graduate School of Medicine at Tokyo Medical and Dental University (M2020-189) and the participating hospital. Data collected from the self-monitoring app and the Fitbit server were automatically stored in a secure database without any personally identifiable information. These datasets were then matched using participant IDs. All participants provided written informed consent prior to participating in the study. The participants received a prepaid card worth JP¥ 3,000 (US $19.36) as a reward for participating in the study, and received a Fitbit used in the study as compensation after the study.

## Results

### Participant Characteristics and MAMP Use Patterns

Table 1 presents the demographic characteristics and conditions related to MAMP use during the study period. The mean age of participants was 47.0 (SD 9.7) years, and the majority were male (6/7, 86%). The average Fitbit wearing rate throughout the study period was 70.1%. The severity of drug dependence assessed using DAST-10 was substantial, with a mean score of 6.0 (SD 1.3). The mean resting heart rate over the study period was 69.8 (SD 8.2) bpm. Emotional status was most frequently reported as “good” (121/308, 39.3%) or “not good” (129/308, 41.9%).

**Table 1.** Participant characteristics, device wearing rate, craving, emotional status, and heart rate during the study period (n=7).

Characterics	Results	
Age (years), mean (SD)	47.0 (9.7)	
Gender (male), n (%)	6 (86)	
Fitbit device wearing rate (%), mean (SD)	70.1 (36.1)	
DAST-10^a^, mean (SD)	6.0 (1.3)	
Craving^b^, mean (SD)	3.9 (3.7)	
Resting heart rate (bpm), mean (SD)	69.8 (8.2)	
Emotion, n (%)^c^		
Good	121 (39.3)	
Not good	129 (41.9)	
Sad	46 (14.9)	
Angry	12 (3.9)	

a DAST-10: Drug Abuse Screening Test 10 (range: 0 to 10).

b Assessed using a numerical rating scale (range: 0 to 10).

c n=308.

Detailed patterns of MAMP use are presented in Table 2 and Multimedia Appendix 1. The mean number of days of MAMP use during the study period was 13.4 (SD 13.9) days over the 56-day study period, ranging from 1 day to 41 days across participants. The number of consecutive days of MAMP use and days of weekend use varied among participants, with mean values of 2.3 (range 1.0-5.9) days and 4.3 (range 0-13) days, respectively. On MAMP use days, the average number of uses was <2 (mean 1.4, SD 0.8). The most frequently reported time of MAMP use depended on the participant; however, it was most commonly in the morning (6-12 AM) or at night (6‐12 PM), accounting for 48.8% (63/129) and 21.7% (28/129) of use events, respectively. Participants who used MAMP frequently (IDs B and C) showed relatively higher resting heart rates than those with less frequent MAMP use.

The completion rate of self-monitoring during the study period and psychological and physiological conditions on MAMP use days are shown in Table 3 and . Throughout the study period, the mean completion rates for self-monitoring of craving and emotional status were 64% (SD 28.4%) and 78.6% (SD 27.3%), respectively. On days when participants used MAMP, emotional status was most frequently reported as “not good” (mean 51%, SD 54.3%) or “sad” (mean 20%, SD 21.3%). The mean craving score was 5.8 (range 2.2-9.8), and resting heart rate averaged 71.3 (range 60.0 to 78.2) bpm; both were higher than the overall averages for the study period. The mean Fitbit wearing rate was 82.1% (range 0%-94.3%), indicating that participants wore the device relatively consistently even on days they used MAMP. However, econsiderable variation in emotional status and craving existed between participants. Sleep duration and frequency tended to be reduced or absent on MAMP-use days or the day afterand Figure S1 in Multimedia Appendix 2).

**Table 2.** Patterns of methamphetamine (MAMP) use among participants (n=7).

Participant ID	MAMP-use days in 56 days (days), n	MAMP use per week (days), mean (SD)	Consecutive MAMP use (days), mean (SD)	Weekend use (days), n (%)	Number of MAMP uses on MAMP use days, mean (SD)	Time period of MAMP use, n (%)				Total MAMP uses, n
						Morning^a^	Afternoon^b^	Night^c^	Midnight^d^	
A	7	0.9 (1.0)	1.0 (0.0)	2 (28.6)	1.1 (0.4)	0 (0)	2 (25)	6 (75)	0 (0)	8
B	22	2.8 (1.7)	2.2 (1.5)	8 (36.4)	1.7 (0.7)	4 (10.5)	11 (28.9)	13 (34.2)	10 (26.3)	38
C	41	5.1 (2.4)	5.9 (4.5)	13 (31.7)	1.2 (0.8)	49 (96.1)	1 (2)	0 (0)	1 (2)	51
D	5	0.6 (0.9)	1.3 (0.4)	0 (0)	1.0 (0.0)	2 (40)	0 (0)	3 (60)	0 (0)	5
E	6	0.8 (0.9)	1.5 (0.5)	4 (66.7)	1.8 (1.2)	3 (27.3)	3 (27.3)	3 (27.3)	2 (18.2)	11
F	12	1.5 (1.2)	1.7 (0.7)	3 (25)	1.3 (0.6)	5 (33.3)	1 (6.7)	2 (13.3)	7 (46.7)	15
G	1	0.1 (0.4)	1.0 (N/A^e^)	0 (0)	1.0 (N/A)	0 (0)	0 (0)	1 (100)	0 (0)	1
All participants	13.4 (13.9)^f^	1.7 (1.7)	2.3 (2.7)	4.3 (26.9)^f^	1.4 (0.8)	63 (48.8)^f^	18 (14)^f^	28 (21.7)^f^	20 (15.5)^f^	129

a 6‐12 AM.

b 12‐6 PM.

c 6‐12 PM.

d 0‐6 AM.

e N/A: not applicable.

f Mean (SD).

**Table 3.** Completion rate of self-monitoring during the study period and device wearing rate, emotional status, craving, and heart rate on days with methamphetamine (MAMP) use (n=7).

Participant ID	Completion of self-monitoring during the study period, n (%)		Fitbit device wearing rate on the day of MAMP use (%), mean (SD)	Emotion, n (%)					Craving^a^, mean (SD)	Resting heart rate (bpm), mean (SD)
	Craving days	Emotional status days		Good	Not good	Sad	Angry	No record		
A	50 (89.3)	49 (87.5)	71.2 (29.3)	3 (42.9)	2 (28.6)	1 (14.3)	1 (14.3)	0 (0)	4.1 (0.6)	69.8 (2.3)
B	32 (57.1)	35 (62.5)	94.3 (11.4)	2 (9.1)	17 (77.3)	2 (9.1)	0 (0)	1 (4.5)	9.0 (1.3)	71.2 (4.2)
C	23 (41.1)	54 (96.4)	88.7 (18.3)	11 (26.8)	3 (7.3)	0 (0)	0 (0)	0 (0)	4.6 (1.3)	78.2 (2.0)
D	28 (50)	55 (98.2)	84.6 (21.1)	0 (0)	2 (40)	3 (60)	0 (0)	0 (0)	4.0 (1.7)	67.3 (3.5)
E	52 (92.9)	48 (85.7)	25.8 (34.3)	0 (0)	2 (33.3)	1 (16.7)	0 (0)	3 (50)	9.8 (0.4)	60.0 (2.6)
F	53 (94.6)	54 (96.4)	77.4 (32.3)	1 (6.7)	4 (26.7)	10 (66.7)	0 (0)	0 (0)	2.2 (2.6)	64.6 (4.7)
G	13 (23.2)	13 (23.2)	0 (N/A^b^)	1 (100)	0 (0)	0 (0)	0 (0)	0 (0)	8.0 (N/A)	N/A
All participants	64 (28.4)^c^	78.6 (27.3)^c^	82.1 (27.9)	18 (19.1)^c^	51 (54.3)^c^	20 (21.3)^c^	1 (1.1)^c^	4 (4.3)^c^	5.8 (3.4)	73.1 (6.4)

a Craving: assessed using a numerical rating scale (range 0 to 10).

b N/A: not applicable.

c Mean (SD).

### Craving and Emotions

Table 4 shows the correlations between psychological and behavioral states and MAMP use. Negative emotions (such as feeling sad or angry) were associated with a higher number of MAMP uses per day. Craving (*r*=0.32, *P*<.001) and resting heart rate (*r*=0.16, *P*<.01) were positively correlated with the number of daily MAMP uses. However, these correlations varied across participants and were not observed in some cases. The Fitbit wearing rate was positively correlated with the number of MAMP uses per day (*r*=0.17, *P*<.001), suggesting that participants tended to wear the device even on days when they used MAMP.

**Table 4.** Correlations between the number of methamphetamine (MAMP) uses per day and emotion, craving, and heart rate on MAMP use days (n=7).

Participant	Correlation with number of MAMP uses per day			
	Emotion^a^, *r*	Craving, *r*	Resting heart rate (bpm), *r*	Device wearing proportion on days of MAMP use, *r*
Participant ID A	0.02	0.37^b^	−0.02	−0.08
Participant ID B	0.00	0.02	−0.11	0.20
Participant ID C	0.20	0.01	−0.05	−0.03
Participant ID D	−0.40^b^	−0.36	−0.20	0.22
Participant ID E	−0.07	0.50^c^	−0.07	−0.43^b^
Participant ID F	−0.63^c^	0.25	0.18	0.03
Participant ID G	0.08	0.03	N/A	−0.06
All participants	−0.12^d^	0.32^c^	0.16^b^	0.17^c^

a Assessed using 4 emotional status categories (good=1, not good=0, sad=−1, angry=−2).

b *P*<.01.

c *P*<.001.

d *P*<.05.

### Heart Rate Response and Interindividual and Intraindividual Differences

**Figure 1.**
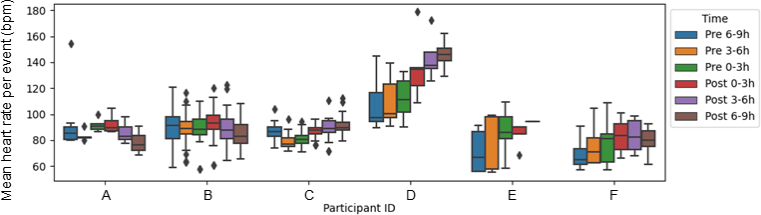
Interindividual differences in methamphetamine use event and heart rate. Box plot of mean heart rate per methamphetamine use event across 6 time intervals for each participant, highlighting interindividual variability and temporal changes in physiological responses. Participant ID G was omitted because only one methamphetamine use record was available, preventing further calculation.

Heart rate tended to increase following MAMP use (Figures1  2, Figure S1 in Multimedia Appendix 2). Table 5 presents the results of a linear mixed effects model evaluating changes in heart rate before and after drug use. A total of 122 observations were included in the analysis, with 5 to 50 observations per participant. The model demonstrated a good fit (log-likelihood=−2626.359). The mean heart rate during the reference period (6‐9 h before drug use) was 89.89 (95% CI 76.88 to 102.89) bpm. At 0 hours to 3 hours postdrug use, mean heart rate increased significantly compared with the reference period (+5.44, 95% CI 3.26 to 7.62 bpm; *P*<.001), and this elevated level was maintained at 3 hours to 6 hours postuse (+5.51, 95% CI 3.04 to 7.98 bpm; *P*<.001). A significant increase was also observed at 6 hours to 9 hours postuse (+3.84, 95% CI 1.27 to 6.41 bpm; *P*=.003). Heart rate during the 0 hours to 3 hours before drug use did not differ significantly from the reference period. However, at 3 hours to 6 hours before drug use, heart rate was significantly lower than during the reference period (−2.71, 95% CI −5.25 to −0.17 bpm; *P*=.04).

Analysis of variance revealed substantial interindividual and intraindividual variability in heart rate responses. Variance was observed at multiple levels: between participants (SD 15.88 bpm), between events within participants (SD 6.76 bpm), and in the residual error (SD 9.80 bpm). The between-participant variance was 250.66, with an intraclass correlation coefficient of 0.64, indicating that approximately 64% of the total variability in heart rate measurements was attributable to differences between individuals. The variance between events within participants was 45.9, accounting for roughly 12% of the total variance. The residual variance was 96.0, suggesting that the same individual may exhibit different heart rate responses to MAMP use on different occasions.

**Figure 2.**
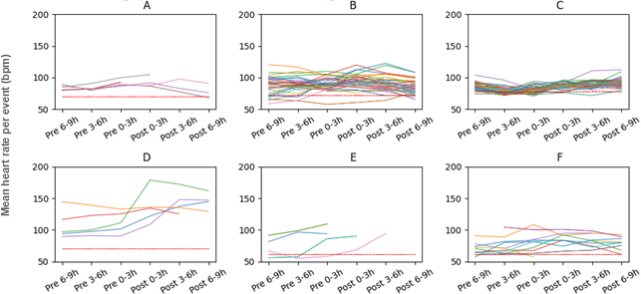
Intraindividual variability in heart rate responses across methamphetamine-use events, shown as changes in mean heart rate across 6 defined intervals surrounding each methamphetamine-use event, represented as a line, for individual participants: (A) participant ID A, (B) participant ID B, (C) participant ID C, (D) participant ID D, (E) participant ID E, and (F) participant ID F. Lines ending prematurely indicate missing heart rate data due to device removal. The red dotted line indicates the average resting heart rate. Participant ID G was omitted because only one methamphetamine use record was available, preventing further calculation.

**Table 5.** Results of a linear mixed effects model on heart rate changes before and after methamphetamine (MAMP) use (n=6; 122 data points included in the analysis): random effect std: participants=15.88 bpm (intraclass correlation coefficient=0.64), events within participants=6.76 bpm (intraclass correlation coefficient=0.12), residual=9.80 bpm.

Time period of MAMP use		Change in heart rate (bpm), coefficient (95% CI)	SE	*t* test	*P* value
Before (pre-; hours)					
6‐9 (Reference)		89.89 (76.88 to 102.89)	6.62	13.58	<.001
3‐6		−2.71 (−5.25 to −0.17)	1.28	−2.10	.04
0‐3		0.13 (−2.37 to 2.65)	1.29	0.11	.92
After (post-; hours)					
0‐3		5.44 (3.26 to 7.62)	1.11	4.9	<.001
3‐6		5.51 (3.04 to 7.98)	1.26	4.38	<.001
6‐9		3.84 (1.27 to 6.41)	1.31	2.94	.003

## Discussion

### Principal Findings

This pilot study presented detailed patterns of MAMP use, along with interindividual and intraindividual variability in physiological and psychological data, using Fitbits and a mobile-based self-monitoring app with individuals with MAMP use disorder. Patterns of MAMP use varied across participants. Craving for MAMP and negative emotions tended to be associated with the number of MAMP uses per day. A persistent increase in heart rate was observed following MAMP use. However, these trends differed between individuals and across MAMP use events. Because the sample size was small and there was considerable variability in the number of MAMP use days across participants, a larger-scale study will be necessary to further examine these associations. In particular, additional analyses are needed to clarify the relationships among craving, emotional states, and MAMP use.

### Variability of MAMP Use Patterns

Among the participants, the frequency of MAMP use varied widely, with the total number of use days ranging from 1 day to 41 days, and consecutive days of use ranging from 1.0 day to 5.9 days. In contrast, the number of MAMP uses per day was relatively consistent among the participants, typically fewer than 2 times per MAMP use day. The participants tended to use MAMP in the mornings and at night on both weekdays and weekends. MAMP is often used to improve performance for daily activities or work, staying awake, helping manage stress, or increasing sexual performance and impulses [38,39]. The participants might also use MAMP when they need to motivate their behavior for specific purposes, whether on weekdays or weekends. This finding is consistent with previous wastewater-based epidemiology studies, which revealed no significant differences in MAMP use between weekdays and weekends [40-42].

Additionally, abstinence following prolonged MAMP use can lead to withdrawal symptoms such as sleep disturbances, difficulty concentrating, fatigue, irritability, agitation, anxiety, sadness, depression, and an inability to engage in normal daily activities [43]. Some participants may struggle to manage these withdrawal symptoms and return to MAMP use. As a result, cycles of binge use and abstinence may occur among individuals with MAMP use disorder. To date, no medications have shown consistent effectiveness for treating MAMP withdrawal [44], and psychosocial treatments remain the primary approach. Cognitive-behavioral therapy (CBT) can be useful for managing withdrawal and craving, supporting sustained abstinence, and facilitating recovery following lapses or relapses [45,46]. Recently, online and computerized forms of CBT have become more widespread and have been shown to be safe, effective, and durable when compared with standard clinician-delivered therapy [47,48]. Incorporating online CBT modules into ecological momentary interventions may be a promising approach for supporting individuals with MAMP use disorder.

### Relationship of Craving and Emotions

On MAMP use days, cravings and negative emotions (eg, feeling sad or unwell) were more frequently reported than in the overall study period. Significant correlations were observed between craving and the number of MAMP use episodes per day, consistent with findings from previous studies. MAMP craving has been shown to persist for at least 5 weeks into abstinence, with individuals being vulnerable to relapse during the first 7 days to 14 days [49] Craving is considered a significant predictor of subsequent MAMP use [50,51]. Negative emotions often intensify cravings, and individuals may be more likely to use MAMP if they do not effectively address these emotions [8,52]. Although the causal relationship between negative emotions and MAMP use was unclear in this study, some participants without adaptive coping strategies may use MAMP as a way to cope with negative emotions. When developing ecological momentary interventions for individuals with MAMP use disorder, incorporating modules for self-monitoring may be helpful to increase awareness of the relationships between emotions, craving, and MAMP use, even during abstinence periods. Additionally, modules for coping with negative emotions may be promising approaches to support MAMP use management.

Overall, tendencies for correlations between MAMP use and craving or negative emotions were observed; however, these relationships and trends varied across the participants. There were some participants without significant correlations between MAMP use and craving or negative emotions. MAMP is often used to reduce withdrawal symptoms from other substances or to cope with pain and discomfort [39]. Additionally, increased tolerance or experiencing a critical life event may lead to increased MAMP use [29]. A previous study suggested that individuals who initiate MAMP use at a later age may do so to “self-medicate” in response to stressful life events [19]. Long-term MAMP users may become less sensitive to cravings due to tolerance. Moreover, some individuals with chronic pain or persistent psychiatric symptoms (eg, depression, anxiety) may use MAMP regardless of craving and emotions. Promoting appropriate and fundamental treatments to address the underlying causes of MAMP use, such as chronic pain and mental health issues, is also essential.

### Heart Rate and Sleep After MAMP Use

After using MAMP, reduction or loss of sleep and a tendency for increased heart rate were observed. Participants with frequent MAMP use exhibited high resting heart rates. MAMP has a strong and relatively long-lasting stimulant effect on the central nervous and cardiovascular systems [20]. Findings from the linear mixed effects model suggest that the cardiovascular effects of MAMP persist for up to 9 hours after use, with an increase of 3 bpm to 5 bpm compared with baseline levels. In line with previous experimental studies [22], heart rate increased following MAMP use. Laboratory-based studies often evaluate short-term cardiovascular responses using standardized doses and predefined routes of administration. In contrast, this study suggests that, in real-world settings, heart rate elevation following MAMP use may persist for up to 9 hours. This study examined naturally occurring MAMP use events under free-living conditions, and despite variability in dosage, routes of administration, and contextual factors, sustained elevations in heart rate were observed. The average heart rates among the participants (approximately 60 bpm to 78 bpm) were within the normal range; however, chronically elevated heart rate can still lead to cardiovascular dysfunction [53-55]. Unexpected harms related to MAMP use, such as stroke, coronary artery disease, and sudden cardiac death, may occur in individuals who use MAMP frequently, even among young adults [19,55,56]. The implementation of an alert system within an ecological momentary intervention app designed to detect and notify individuals of sudden heart rate increases or arrhythmias may contribute to the prevention of cardiovascular events.

### Interindividual and Intraindividual Differences in Heart Rate Response

Findings from the analysis of varianceA indicated that increases in heart rate were attributable to both individual differences and MAMP use events. Approximately 64% of the variance in heart rate was due to differences between participants, suggesting significant heterogeneity in individual responses to MAMP use. Previous studies have suggested that gender differences and genetic variations may help explain individual differences in responses to MAMP use [57,58]. Identifying individuals who exhibit substantial increases in heart rate following MAMP use and providing real-time notifications of elevated heart rate may be a useful strategy for risk management. In contrast, variance between events within individuals accounted for approximately 12% of the total variance, indicating that even the same individual may experience different cardiovascular responses to MAMP on different occasions. These findings have important clinical implications, emphasizing that drug effects are not consistent, even within individuals. These differences may be influenced by the duration of MAMP effects and bioavailability, which are affected by various factors such as the route of administration (eg, injection, smoking) and the amount of MAMP used [20,43]. Even among individuals with relatively mild cardiovascular responses, any changes in the administration or dosage of use should be approached with caution. To comprehensively understand variability in heart rate response to MAMP use, it is essential to consider both interindividual and intraindividual variability.

### Implications for Future Research

As a pilot study, these findings provide important insights for the design of future EMA-based research on MAMP use. Specifically, the results can inform the optimization of EMA protocols, including the timing and frequency of pre- and post-MAMP use. In this study, we were unable to determine causal relationships between psychological states and MAMP use, as craving and emotional status were assessed only once per day. More frequent self-monitoring may enable more precise assessment of craving and emotional states and help elucidate their temporal associations with MAMP use. Such refinements may enhance the ability to capture within-person dynamics and identify high-risk periods that could be targeted in future intervention studies. Furthermore, the study findings may inform the development of personalized interventions that account for both between-person and within-person variability in associations with MAMP use and physiological responses or psychological states.

### Limitations

This pilot study has several limitations. First, self-monitoring was conducted only once per day, which limited our ability to evaluate causal relationships between craving, emotions, and MAMP use. In our ongoing study, self-monitoring is conducted 3 times per day using an EMA approach to address this limitation. Second, we did not assess nor adjust for potential confounding factors such as smoking habits, night shift work, or the use of alcohol and other substances, which may influence heart rate and sleep patterns. Although other drug use, such as opioids, was rare among participants in this study, these variables are assessed in our ongoing research. Third, the sample size was small, and the participants were only individuals who received outpatient treatment. This may have biased the results. Further research with a larger sample and individuals with more active MAMP use is needed, along with strategies to improve adherence to wearable devices and response rates to self-monitoring prompts. Last, we did not collect data on the route of administration or the quantity of MAMP. To more precisely evaluate heart rate responses, information on how MAMP is used and in what amounts may be important.

### Conclusions

This pilot study captured psychological status and physiological responses related to MAMP use, using an EMA framework. Patterns of MAMP use varied among individuals with MAMP use disorder. Overall, craving and negative emotions were correlated with the frequency of MAMP use per day; however, this relationship differed across participants. Similar physiological responses were commonly observed following MAMP use, including persistent increases in heart rate and sleep disturbances, which can lead to cardiovascular dysfunction. Heart rate increased significantly by approximately 3 bpm to 5 bpm compared with the period before MAMP use and remained elevated for up to 9 hours. However, increases in heart rate were explained not only by MAMP use events but also by individual differences. To better understand the individualized predictors for use and physiological responses to use, it is essential to assess both interindividual and intraindividual variability. The findings should be interpreted as exploratory and reflective of within-person patterns rather than representative of group-level trends. Further studies with larger and more diverse samples are needed to assess generalizability.

## Supplementary material

10.2196/73790 Multimedia Appendix 1 Heatmaps visualizing daily physiological responses and psychological conditions, with METH Use Event reflecting the number of drug use events, Weekend indicating whether a day is a weekend (weekdays are shown in lighter shades), and emotions color-coded as follows: angry, dark red; sad, light red; not good, light blue; good, dark blue. Darker colors for METH Use Event, Device Wearing Rate, Sleep Count, Total Main Sleep Time, and Resting Heart Rate indicate higher frequency of drug use, longer durations of device use, a greater number of sleep episodes, longer sleep duration, and a higher resting heart rate, respectively. METH: methamphetamine.

10.2196/73790 Multimedia Appendix 2 Physiological data collected during the measurement period, with each row representing 1 week, starting from Monday.

## Abbreviations

API: application programming interface
CBT: cognitive-behavioral therapy
DAST: Drug Abuse Screening Test
EMA: ecological momentary assessment
MAMP: methamphetamine
SUD: substance use disorder

## References

[1] Stern E, Micoulaud Franchi JA, Dumas G (2023). How can digital mental health enhance psychiatry?. Neuroscientist.

[2] Bond RR, Mulvenna MD, Potts C, O’Neill S, Ennis E, Torous J (2023). Digital transformation of mental health services. Npj Ment Health Res.

[3] Smith KA, Blease C, Faurholt-Jepsen M (2023). Digital mental health: challenges and next steps. BMJ Ment Health.

[4] Bertz JW, Epstein DH, Preston KL (2018). Combining ecological momentary assessment with objective, ambulatory measures of behavior and physiology in substance-use research. Addict Behav.

[5] Jordan HR, Sahni S, Ahmed MM (2023). A comprehensive literature review of digital health interventions in the treatment of substance use disorder with special focus on mobile applications. Cureus.

[6] Boumparis N, Schaub MP (2022). Recent advances in digital health interventions for substance use disorders. Curr Opin Psychiatry.

[7] O’Logbon J, Wickersham A, Williamson C, Leightley D (2024). The effectiveness of digital health technologies for reducing substance use among young people: a systematic review & meta-analysis. J Ment Health.

[8] Serre F, Fatseas M, Swendsen J, Auriacombe M (2015). Ecological momentary assessment in the investigation of craving and substance use in daily life: a systematic review. Drug Alcohol Depend.

[9] Lattie EG, Adkins EC, Winquist N, Stiles-Shields C, Wafford QE, Graham AK (2019). Digital mental health interventions for depression, anxiety, and enhancement of psychological well-being among college students: systematic review. J Med Internet Res.

[10] Stevenson BL, Kunicki ZJ, Brick L, Blevins CE, Stein M, Abrantes AM (2022). Using ecological momentary assessments and Fitbit data to examine daily associations between physical activity, affect and alcohol cravings in patients with alcohol use disorder. Int J Behav Med.

[11] De Rosa O, Menghini L, Kerr E (2025). Exploring the relationship between sleep patterns, alcohol and other substances consumption in young adults: insights from wearables and mobile surveys in the National Consortium on Alcohol and NeuroDevelopment in Adolescence (NCANDA) cohort. Int J Psychophysiol.

[12] Oesterle TS, Karpyak VM, Coombes BJ (2022). Systematic review: wearable remote monitoring to detect nonalcohol/nonnicotine-related substance use disorder symptoms. Am J Addict.

[13] Votaw VR, Witkiewitz K (2021). Motives for substance use in daily life: a systematic review of studies using ecological momentary assessment. Clin Psychol Sci.

[14] Davis-Martin RE, Alessi SM, Boudreaux ED (2021). Alcohol use disorder in the age of technology: a review of wearable biosensors in alcohol use disorder treatment. Front Psychiatry.

[15] Carreiro S, Newcomb M, Leach R, Ostrowski S, Boudreaux ED, Amante D (2020). Current reporting of usability and impact of mHealth interventions for substance use disorder: a systematic review. Drug Alcohol Depend.

[16] Abdelaal Y, Aupetit M, Baggag A, Al-Thani D (2024). Exploring the applications of explainability in wearable data analytics: systematic literature review. J Med Internet Res.

[17] Sabry F, Eltaras T, Labda W, Alzoubi K, Malluhi Q (2022). Machine learning for healthcare wearable devices: the big picture. J Healthc Eng.

[18] Vos G, Trinh K, Sarnyai Z, Rahimi Azghadi M (2023). Generalizable machine learning for stress monitoring from wearable devices: a systematic literature review. Int J Med Inform.

[19] Paulus MP, Stewart JL (2020). Neurobiology, clinical presentation, and treatment of methamphetamine use disorder: a review. JAMA Psychiatry.

[20] Courtney KE, Ray LA (2014). Methamphetamine: an update on epidemiology, pharmacology, clinical phenomenology, and treatment literature. Drug Alcohol Depend.

[21] Hart CL, Gunderson EW, Perez A (2008). Acute physiological and behavioral effects of intranasal methamphetamine in humans. Neuropsychopharmacol.

[22] Fleury G, De La Garza R, Mahoney JJ, Evans SE, Newton TF (2008). Predictors of cardiovascular response to methamphetamine administration in methamphetamine‐dependent individuals. American J Addict.

[23] Perez AY, Kirkpatrick MG, Gunderson EW (2008). Residual effects of intranasal methamphetamine on sleep, mood, and performance. Drug Alcohol Depend.

[24] Mazinani SM, Sadeghi Bajestani G (2019). PSG dynamic changes in methamphetamine abuse using recurrence quantification analysis. IIUMEJ.

[25] Rezaei-Ardani A, Rezaei-Talab F, Afshari-Saleh L, Asad-Pour H, Amjadi-Goojgi Z (2021). Polysomnographic survey of sleep architecture in patients with methamphetamine dependence during remission. Sleep Sci.

[26] Reback CJ, Rünger D, Fletcher JB, Swendeman D (2018). Ecological momentary assessments for self-monitoring and counseling to optimize methamphetamine treatment and sexual risk reduction outcomes among gay and bisexual men. J Subst Abuse Treat.

[27] Sun Y, Kargarandehkordi A, Slade C (2024). Personalized deep learning for substance use in Hawaii: protocol for a passive sensing and ecological momentary assessment study. JMIR Res Protoc.

[28] Shimane T, Imamura A, Ikeda K (2015). Reliability and validity of the Japanese version of the DAST-20. Nihon Arukoru Yakubutsu Igakkai Zasshi.

[29] Yudko E, Lozhkina O, Fouts A (2007). A comprehensive review of the psychometric properties of the Drug Abuse Screening Test. J Subst Abuse Treat.

[30] Takano A, Ono K, Nozawa K (2023). Wearable sensor and mobile app-based mHealth approach for investigating substance use and related factors in daily life: protocol for an ecological momentary assessment study. JMIR Res Protoc.

[31] Fitbit inspire 2 user manual version 1.0. Fitbit Inc.

[32] Mendelson J, Uemura N, Harris D (2006). Human pharmacology of the methamphetamine stereoisomers. Clin Pharmacol Ther.

[33] Chevance G, Golaszewski NM, Tipton E (2022). Accuracy and precision of energy expenditure, heart rate, and steps measured by combined-sensing Fitbits against reference measures: systematic review and meta-analysis. JMIR Mhealth Uhealth.

[34] Fuller D, Colwell E, Low J (2020). Reliability and validity of commercially available wearable devices for measuring steps, energy expenditure, and heart rate: systematic review. JMIR Mhealth Uhealth.

[35] Haghayegh S, Khoshnevis S, Smolensky MH, Diller KR, Castriotta RJ (2019). Accuracy of wristband Fitbit models in assessing sleep: systematic review and meta-analysis. J Med Internet Res.

[36] Gass JC, Funderburk JS, Shepardson R, Kosiba JD, Rodriguez L, Maisto SA (2021). The use and impact of self-monitoring on substance use outcomes: a descriptive systematic review. Subst Abus.

[37] Bates D, Mächler M, Bolker BM, Walker SC (2015). Fitting linear mixed-effects models using lme4. J Stat Softw.

[38] O’Donnell A, Addison M, Spencer L (2019). Which individual, social and environmental influences shape key phases in the amphetamine type stimulant use trajectory? A systematic narrative review and thematic synthesis of the qualitative literature. Addiction.

[39] Hançer Tok H, Tokur Kesgin M (2024). Reasons for using methamphetamine: systematic review. Arch Psychiatr Nurs.

[40] Li W, Lu J, Zhao H, Zhao J, Yan Y, Xu Y (2024). International Workers’ Day: consumption patterns of morphine, codeine, and methamphetamine in urban and rural areas based on wastewater-based epidemiology. Environ Toxicol Chem.

[41] Lim HH, Cha HJ, Oh JE (2024). Assessment of illicit drug use in Seoul, the capital of South Korea for 21 days by wastewater-based epidemiology. Sci Total Environ.

[42] Ter Laak TL, Emke E, Benschop A, Nabben T, Béen F (2022). Triangulating Amsterdam’s illicit stimulant use trends by wastewater analysis and recreational drug use monitoring. Forensic Sci Int.

[43] Ciccarone D, Shoptaw S (2022). Understanding stimulant use and use disorders in a new era. Med Clin North Am.

[44] Shoptaw SJ, Kao U, Heinzerling K, Ling W (2009). Treatment for amphetamine withdrawal. Cochrane Database Syst Rev.

[45] Minozzi S, Saulle R, Amato L, Traccis F, Agabio R (2024). Psychosocial interventions for stimulant use disorder. Cochrane Database Syst Rev.

[46] AshaRani PV, Hombali A, Seow E, Ong WJ, Tan JH, Subramaniam M (2020). Non-pharmacological interventions for methamphetamine use disorder: a systematic review. Drug Alcohol Depend.

[47] Magill M, Kiluk BD, Ray LA (2023). Efficacy of cognitive behavioral therapy for alcohol and other drug use disorders: is a one-size-fits-all approach appropriate?. Subst Abuse Rehabil.

[48] Kiluk BD, Nich C, Buck MB (2018). Randomized clinical trial of computerized and clinician-delivered CBT in comparison with standard outpatient treatment for substance use disorders: primary within-treatment and follow-up outcomes. Am J Psychiatry.

[49] Zorick T, Nestor L, Miotto K (2010). Withdrawal symptoms in abstinent methamphetamine-dependent subjects. Addiction.

[50] Galloway GP, Singleton EG, The Methamphetamine Treatment Project Corporate Authors (2009). How long does craving predict use of methamphetamine? Assessment of use one to seven weeks after the assessment of craving: craving and ongoing methamphetamine use. Subst Abuse.

[51] Hartz DT, Frederick-Osborne SL, Galloway GP (2001). Craving predicts use during treatment for methamphetamine dependence: a prospective, repeated-measures, within-subject analysis. Drug Alcohol Depend.

[52] Sayette MA (2016). The role of craving in substance use disorders: theoretical and methodological issues. Annu Rev Clin Psychol.

[53] Kaye S, McKetin R, Duflou J, Darke S (2007). Methamphetamine and cardiovascular pathology: a review of the evidence. Addiction.

[54] Tobolski J, Sawyer DB, Song SJ, Afari ME (2022). Cardiovascular disease associated with methamphetamine use: a review. Heart Fail Rev.

[55] Grassi S, Vaiano F, Dimitrova A (2025). Fatal intoxications and inherited cardiac disorders in the young: where to draw the line?. Int J Legal Med.

[56] Schwarzbach V, Lenk K, Laufs U (2020). Methamphetamine-related cardiovascular diseases. ESC Heart Fail.

[57] Haile CN, Kosten TR, Kosten TA (2009). Pharmacogenetic treatments for drug addiction: cocaine, amphetamine and methamphetamine. Am J Drug Alcohol Abuse.

[58] Dluzen DE, Liu B (2008). Gender differences in methamphetamine use and responses: a review. Gend Med.

