# Supplementary figures and images for: Individual Variability in Physiological Responses and Psychological Conditions Associated With Methamphetamine Use: Pilot Ecological Momentary Assessment Study Using a Wearable Device and Self-Monitoring Mobile App
Source: JMIR Form Res. 2026 Mar 2;10:e73790. doi: 10.2196/73790 (PMC12954691; doi:10.2196/73790)

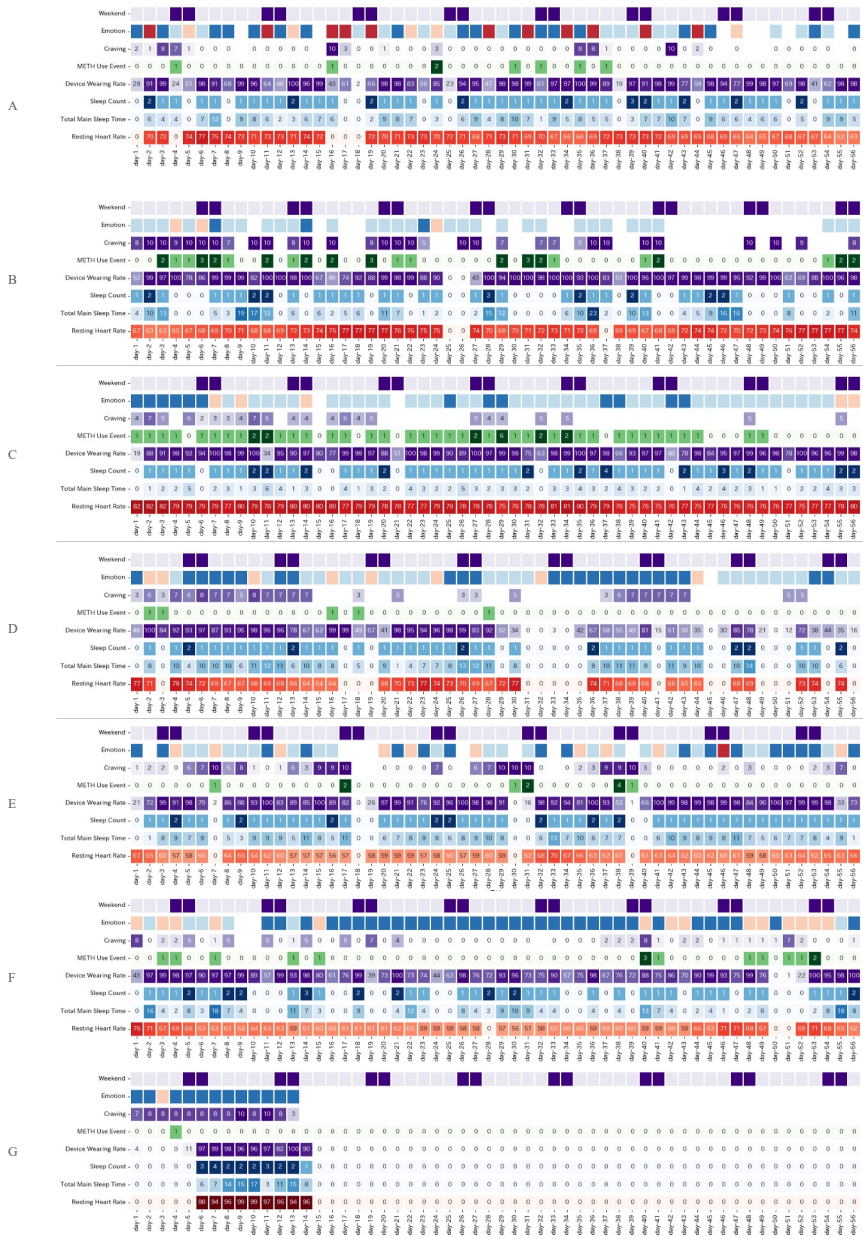

Supplement: Multimedia Appendix 1 [file formative-v10-e73790-s001.pdf]
