# Supplementary material for: Individual Variability in Physiological Responses and Psychological Conditions Associated With Methamphetamine Use: Pilot Ecological Momentary Assessment Study Using a Wearable Device and Self-Monitoring Mobile App
Source: JMIR Form Res. 2026 Mar 2;10:e73790. doi: 10.2196/73790 (PMC12954691; doi:10.2196/73790)
Supplement: Multimedia Appendix 2 [file formative-v10-e73790-s002.pptx]

## Slide 1
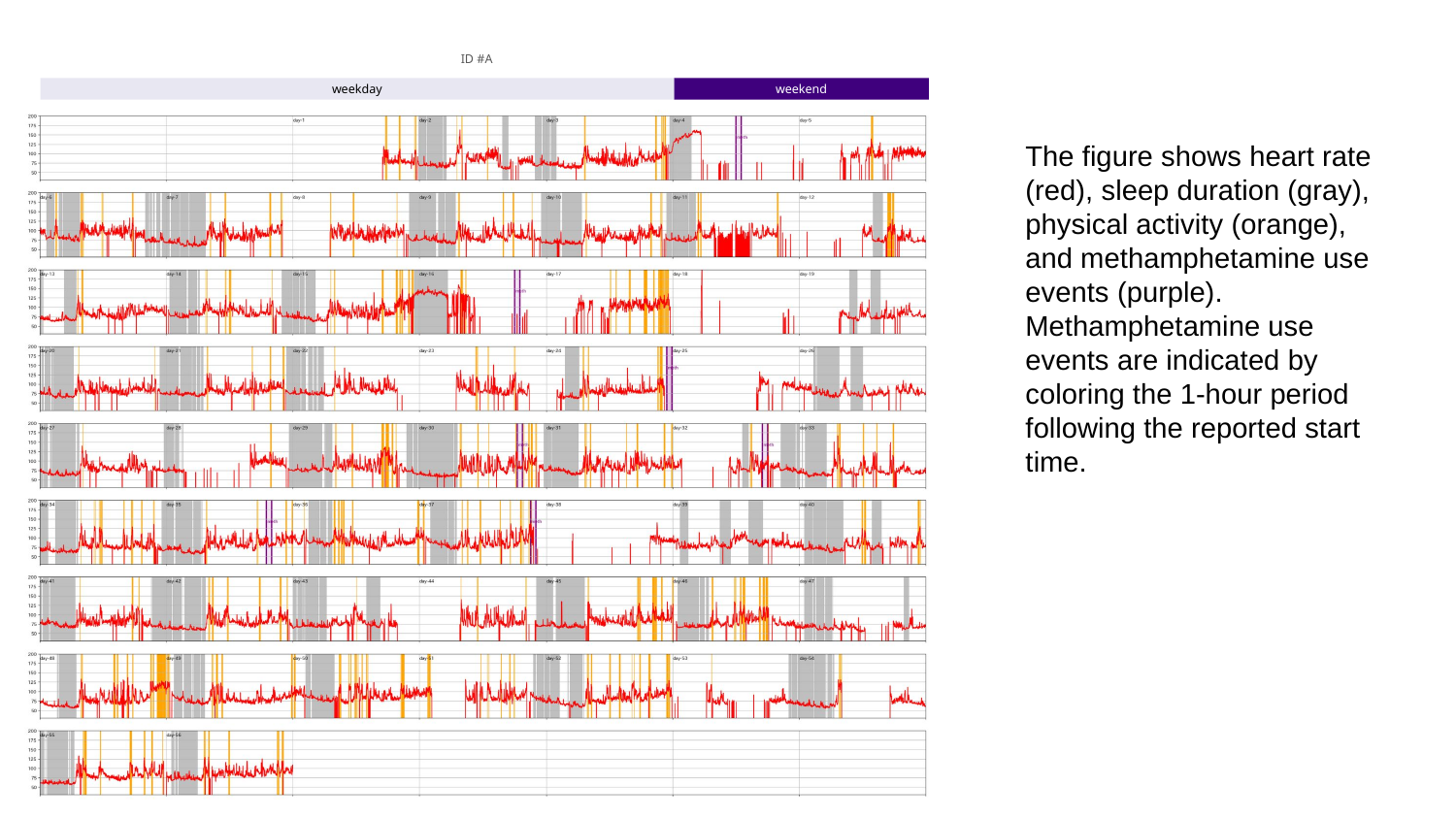

ID #A
weekday
weekend
The figure shows heart rate (red), sleep duration (gray), physical activity (orange), and methamphetamine use events (purple). Methamphetamine use events are indicated by coloring the 1-hour period following the reported start time.

## Slide 2
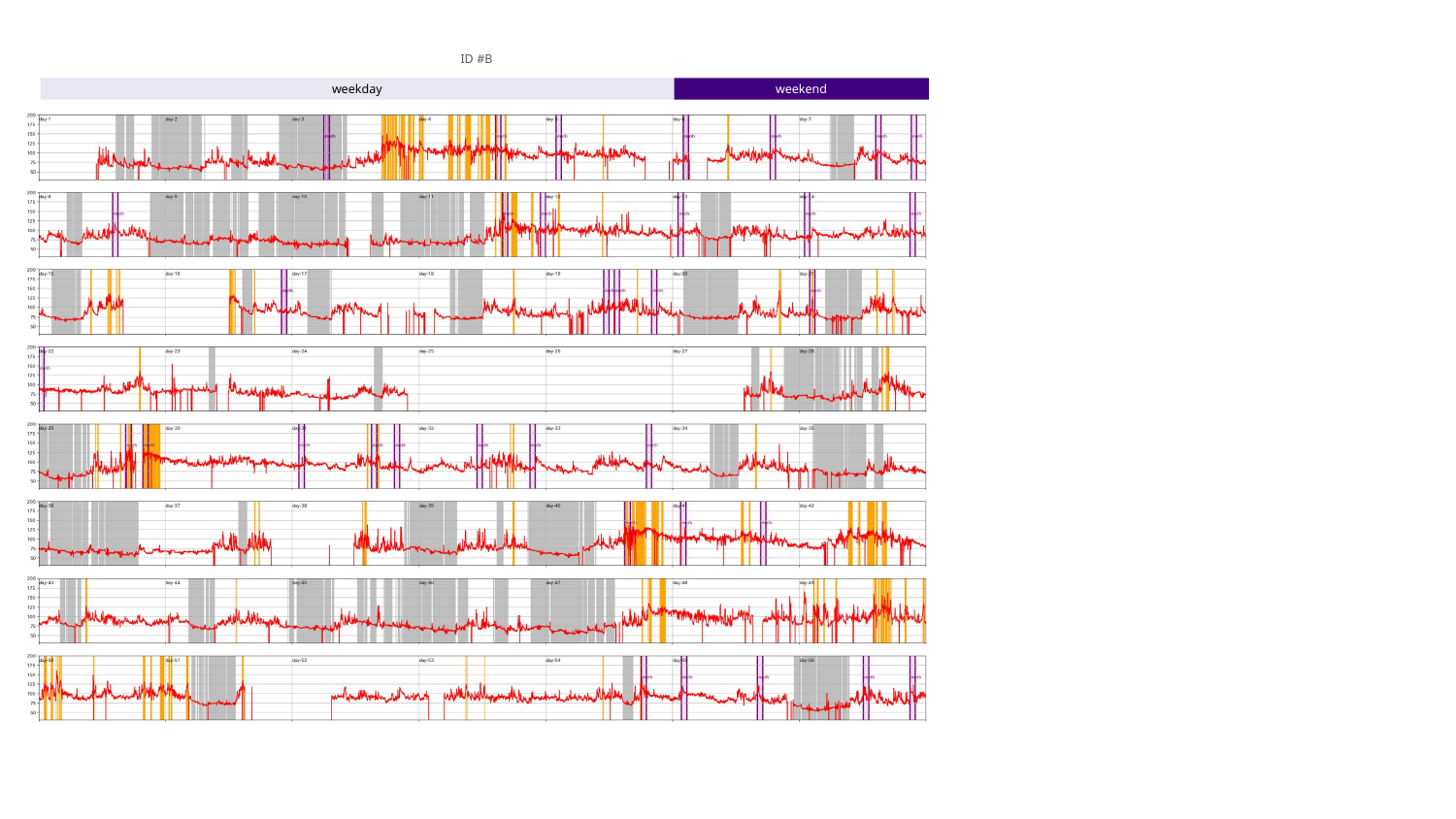

ID #B
weekday
weekend

## Slide 3
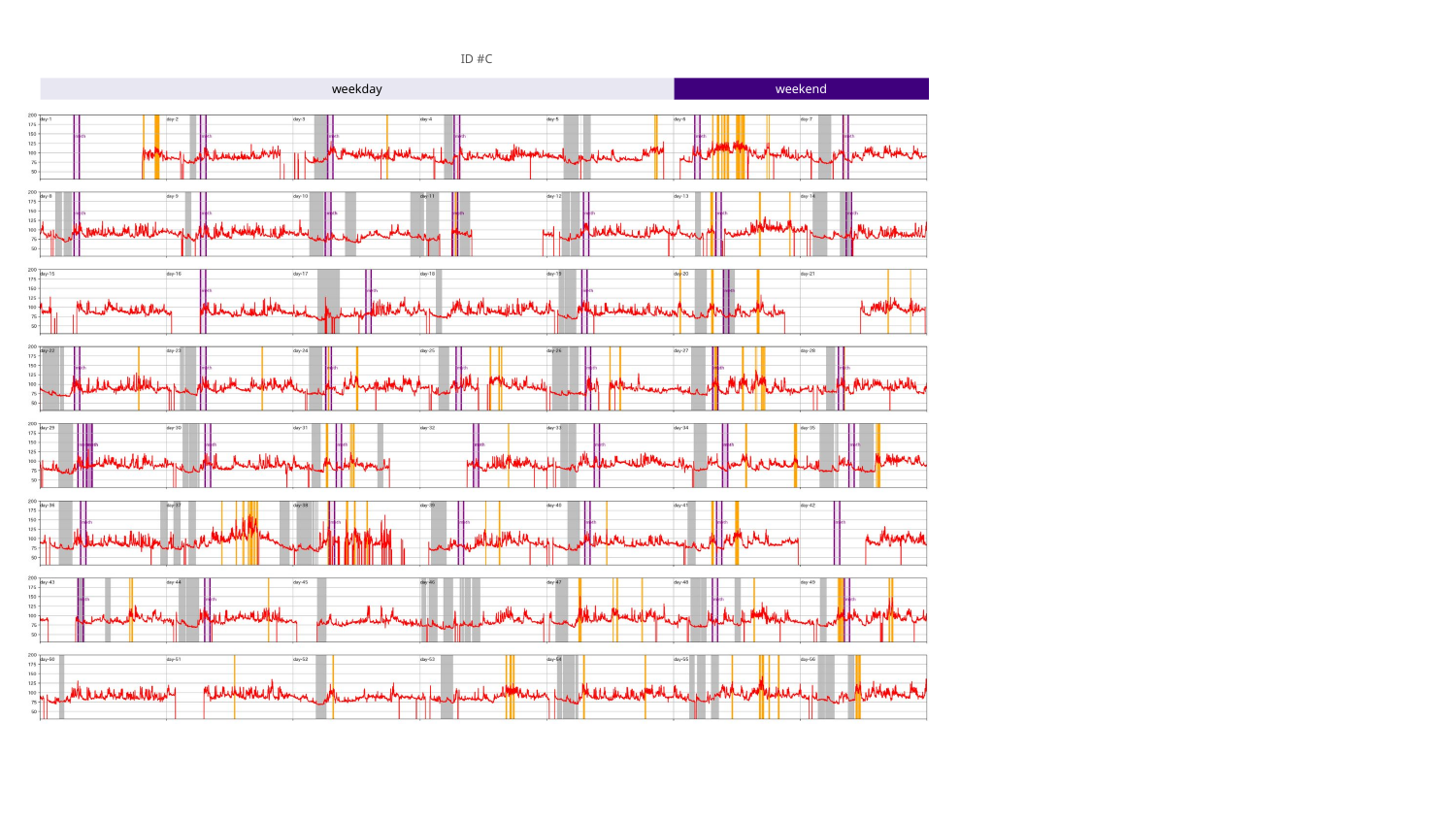

ID #C
weekday
weekend

## Slide 4
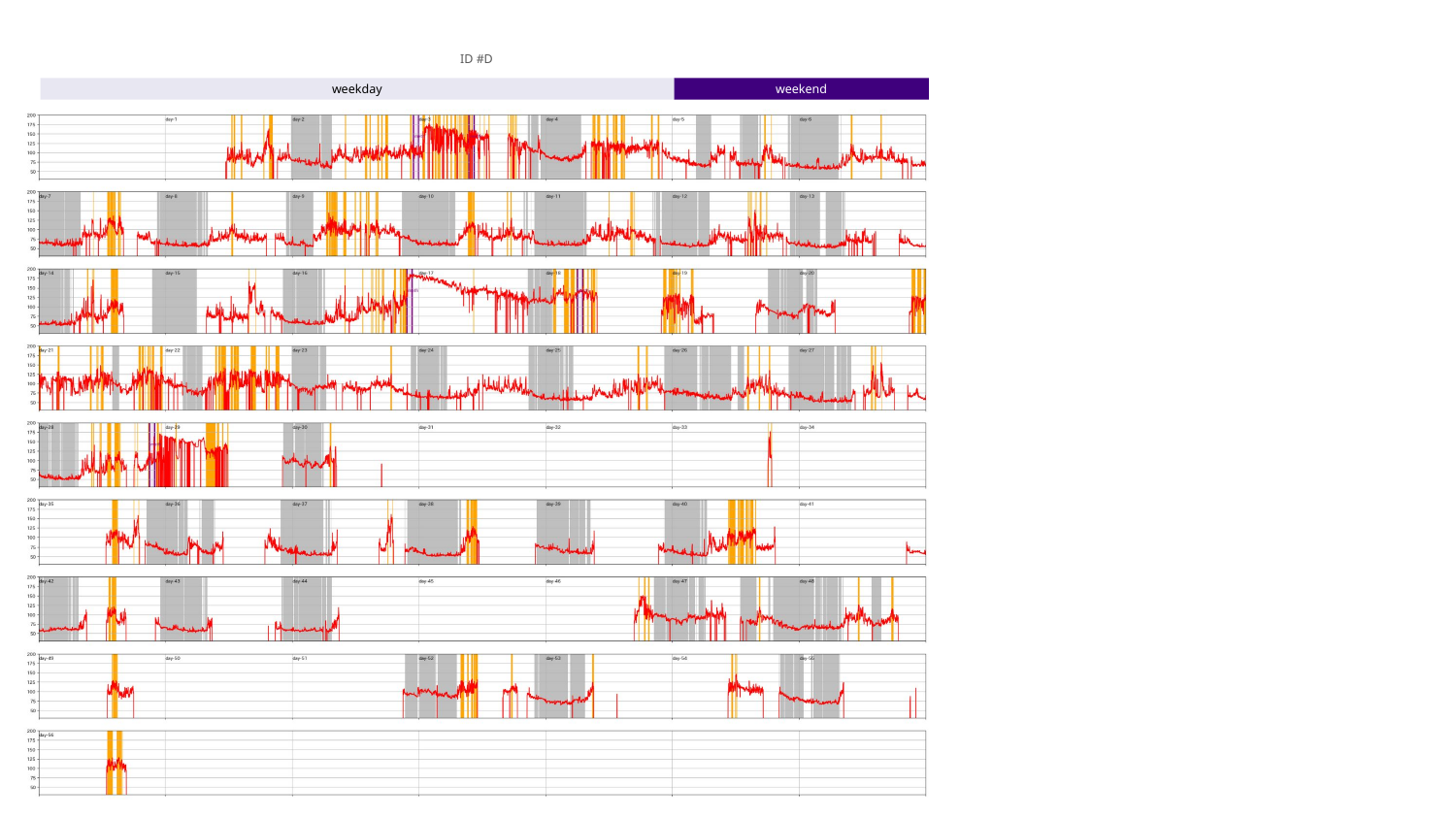

ID #D
weekday
weekend

## Slide 5
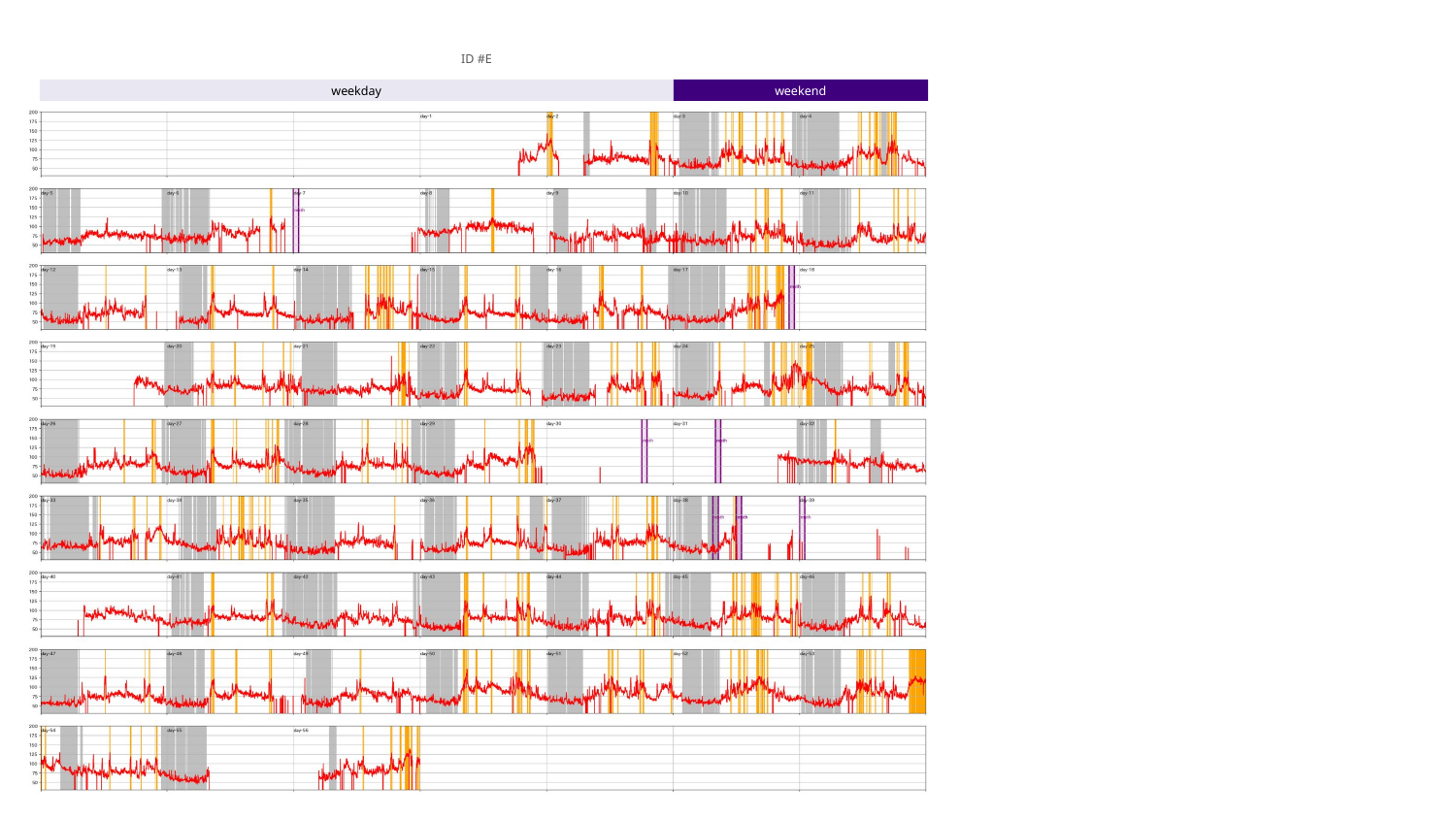

ID #E
weekday
weekend

## Slide 6
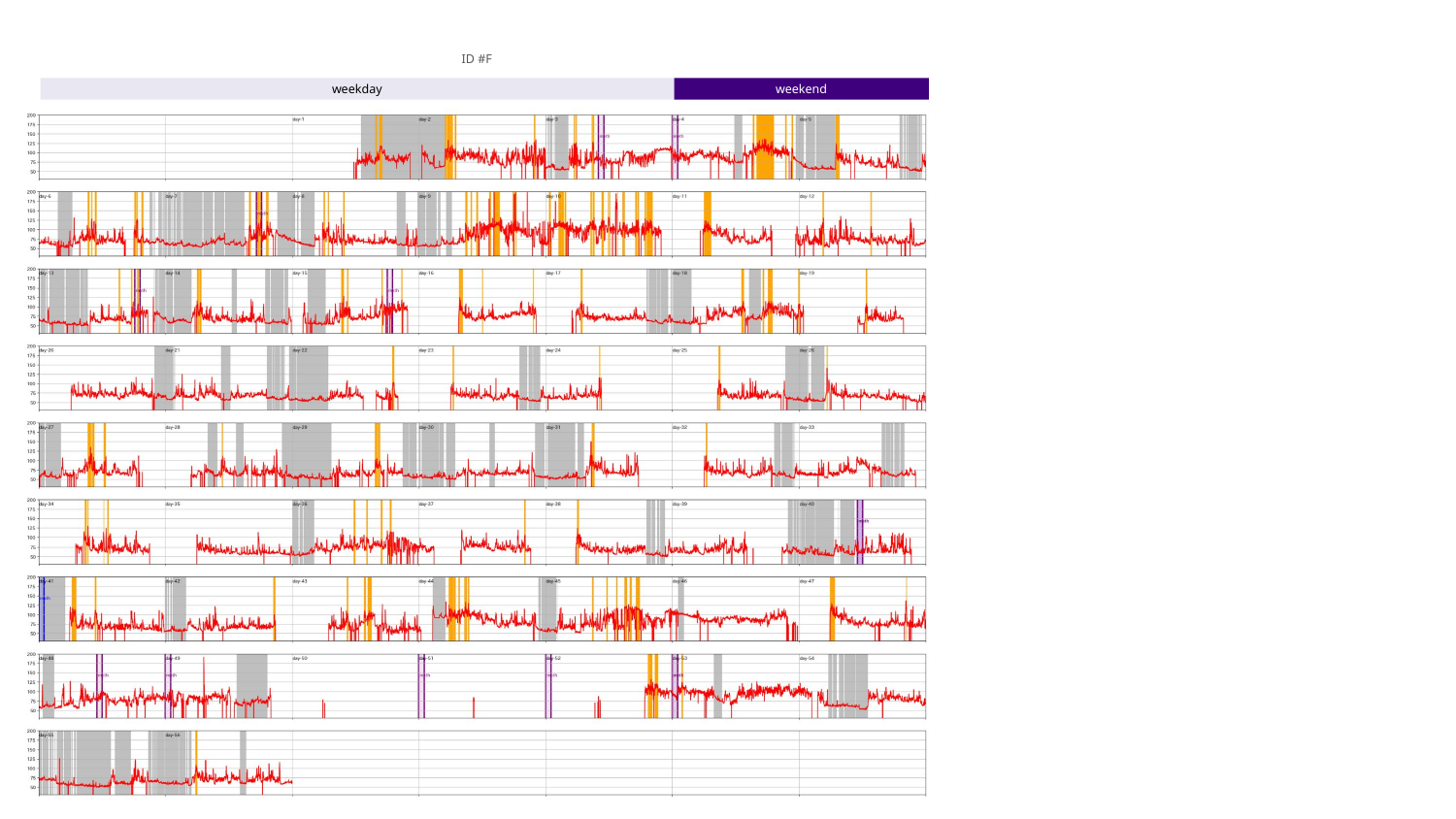

ID #F
weekday
weekend

## Slide 7
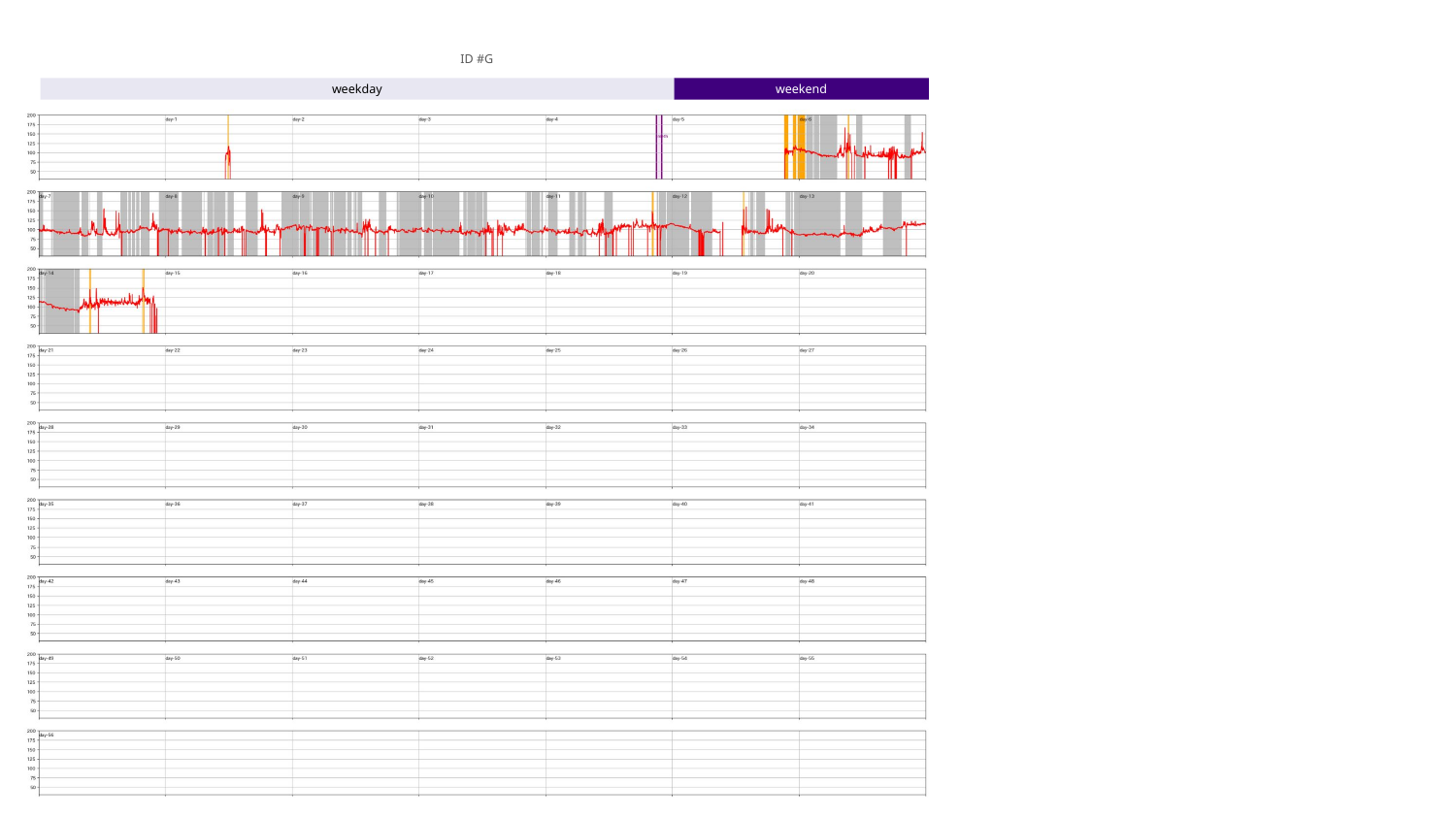

ID #G
weekday
weekend
